# Gap Junctions May Have A Computational Function In The Cerebellum: A Hypothesis

**DOI:** 10.1007/s12311-024-01680-3

**Published:** 2024-03-18

**Authors:** Mike Gilbert, Anders Rasmussen

**Affiliations:** 1 School of Psychology, College of Life and Environmental Sciences, University of Birmingham, B15 2TT, Birmingham, UK; 2 Department of Experimental Medical Science, Lund University, BMC F10, 22184 Lund, Sweden

**Keywords:** Hypothesis, Computational, Cerebellum, Gap Junction, Golgi Cell, Granule Cell

## Abstract

In the cerebellum, granule cells make parallel fibre contact on (and excite) Golgi cells and Golgi cells inhibit granule cells, forming an open feedback loop. Parallel fibres excite Golgi cells synaptically, each making a single contact. Golgi cells inhibit granule cells in a structure called a glomerulus almost exclusively by GABA spillover acting through extrasynaptic GABA_A_ receptors. Golgi cells are connected dendritically by gap junctions. It has long been suspected that feedback contributes to homeostatic regulation of parallel fibre signals activity, causing the fraction of the population that are active to be maintained at a low level. We present a detailed neurophysiological and computationally-rendered model of functionally grouped Golgi cells which can infer the density of parallel fibre signals activity and convert it into proportional modulation of inhibition of granule cells. The conversion is unlearned and not actively computed; rather, output is simply the computational effect of cell morphology and network architecture. Unexpectedly, the conversion becomes more precise at low density, suggesting that self-regulation is attracted to sparse code, because it is stable. A computational function of gap junctions may not be confined to the cerebellum.

## Introduction

Golgi cells are large interneurons whose cell bodies lie in the inner layer, the granular layer, of the cerebellar cortex, most of them concentrated immediately below the Purkinje cell layer. 4–10 dendrites emerge from Golgi cell body of which 2–4 turn upward and give rise to apical dendrites [1]. Apical dendrites traverse the outer layer of the cerebellar cortex, the molecular layer, branching sparsely. Golgi cells receive excitatory input to apical dendrites from parallel fibres, the axons of granule cells, and in turn inhibit granule cells, forming an open feedback loop (the population of granule cells that excite them and the population they inhibit are largely different). Parallel fibres lie parallel to the pial surface and each other, and make contact in passing. Inhibition of granule cells is exclusively by Golgi cells, in a structure termed a glomerulus, ensheathed by a semi-permeable membrane which restricts neurotransmitter diffusion.

We propose that Golgi cells (working together in ensembles) implement a computational conversion that can be tested by simulation *in silico*. The function of the conversion is to turn the level of parallel fibre activity (meaning, the fraction that are active in the general population) into proportional modulation of the inhibition of granule cells, so that an increase or decrease of the fraction causes proportionally stronger or weaker inhibition, respectively.

It is a long-standing idea that parallel fibre activity might be maintained at a low [2] or fixed [3] level by feedback via Golgi cells, but has lacked a detailed mechanism. We explored the idea that Golgi cells form functionally-defined ensembles and that the ‘computation’ by an ensemble is the automated consequence of a combination of cell morphology and ensemble architecture.

Speaking generally, rate information transitions through multiple biophysical steps even in transit through a single neuron. Normally, these are measured on different scales. Scales are arbitrary *per se* but bring consistency to data collection. We float the idea that on a normalised scale, the data coded at each step are related by straightforward functions, so that they can provide the physiological form of mathematical operations, and therefore add steps (but not necessarily sophistication) to neural computations. This is counter to the appearance of the hard-to-see relationship of biophysical measurements. However, it is consistent with accumulating evidence of linear transmission of rate information. If we take this step, there is – we further propose – a computational effect of ensemble anatomy: the ensemble conversion.

There are three steps of the ensemble conversion. At each step, multiple computations are performed in parallel. We give the first step the most attention, but it does not have more functional importance than the others.

In step one, apical dendrites receive contact from a number of active parallel fibres with a distributed probability. The probability distribution depends on the density of parallel fibre activity, and density reflects the percentage of active cells, assuming active cells are a random sample of the population at large. Dendritic charge is modulated by a combination of synaptically-activated influx and passive charge equalisation through gap junctions [4, 5]. The computational function of gap junctions is that they create directly gap-junction-connected groups. Groups effectively randomly sample a nominal population of values implied by the probability distribution, with replacement. Equalisation provides the physiological equivalent of taking the sample means, generating a new population of values that has a reliable statistical relationship with the implied data, by the central limit theorem.

During behaviour (in this model), dendritic depolarisation is sustained despite the fact that apical dendrites individually receive at any time a modest number of co-active inputs. In the second step, dendritic charge is integrated at the Golgi cell soma. Charge transfer to the soma is passive so somatic membrane potential varies proportionally with the mean of dendritic charge. There is a linear conversion of somatic charge to firing of the cell.

Each glomerulus receives innervation from a random sample of Golgi cells afferent to a field (as defined in section 3.2.2). In the third step, this provides a second layer of random sampling (this time, of Golgi cell rates). Granule cell inhibition is almost exclusively by GABA spillover. Intraglomerular GABA concentration varies proportionally with the mean of afferent rates, and inhibition of granule cells varies proportionally with GABA concentration.

As the conversion is a computational effect of ensemble architecture, we can replicate it *in silico* in high resolution because a rich anatomical literature provides computationally-relevant parameters (and the values they take, usually a range). Our aims are: (1) To propose a computational interpretation of the anatomy of the pathway granule cell-Golgi cell-granule cell. We hypothesise that there is a physiologically plausible and statistically reliable conversion of the percentage of parallel fibres that are active in the general population and modulation of inhibition of granule cells. (2) To test the ideas with a computer simulation. We find that the mechanism is computationally viable, i.e., the proposals are a candidate to explain the evidence. We find in addition that the percentage of active parallel fibres is an important contributor to its own regulation, because a low percentage is more stable, so activity is attracted to that level, arguing for a sparse code.

An important note on the use of ‘random’. Part of our proposition is that cell morphologies and network connections are part of a physiological design that exploits statistical effects of random sampling. Physiology uses an anatomical facsimile of truly random sampling to closely approximate the same result. Outside the simulation, when we refer to random we mean a biological proxy.

## Evidence for a Substrate of Linear Relationships

The purpose of the section is to provide a basis in evidence for the proposal that data coded in a chain of biophysical forms could be related by straightforward computational functions. Relationships are not quantified in this section.

### Apical Dendritic Membrane Potential is Proportional to a Count of Active Inputs to a Gap Junction Group

Individual excitatory postsynaptic currents are modest and brief, smaller than mossy fibre charge transfer, consistent with a single site of contact [6, 7]. In slices, single weak parallel fibre signals^1^ generate weak dendritic signals which attenuate with distance [7]. However, there is charge transfer between Golgi cells through apical dendritic gap junctions [4, 5, 8]. Gap junctions counteract attenuation of dendritic charge transfer to the soma by allowing charge admitted following synaptic activation to flow into the dendrites of connected Golgi cell neighbours [9]. Charge transfer is passive.

**Fig 1:**
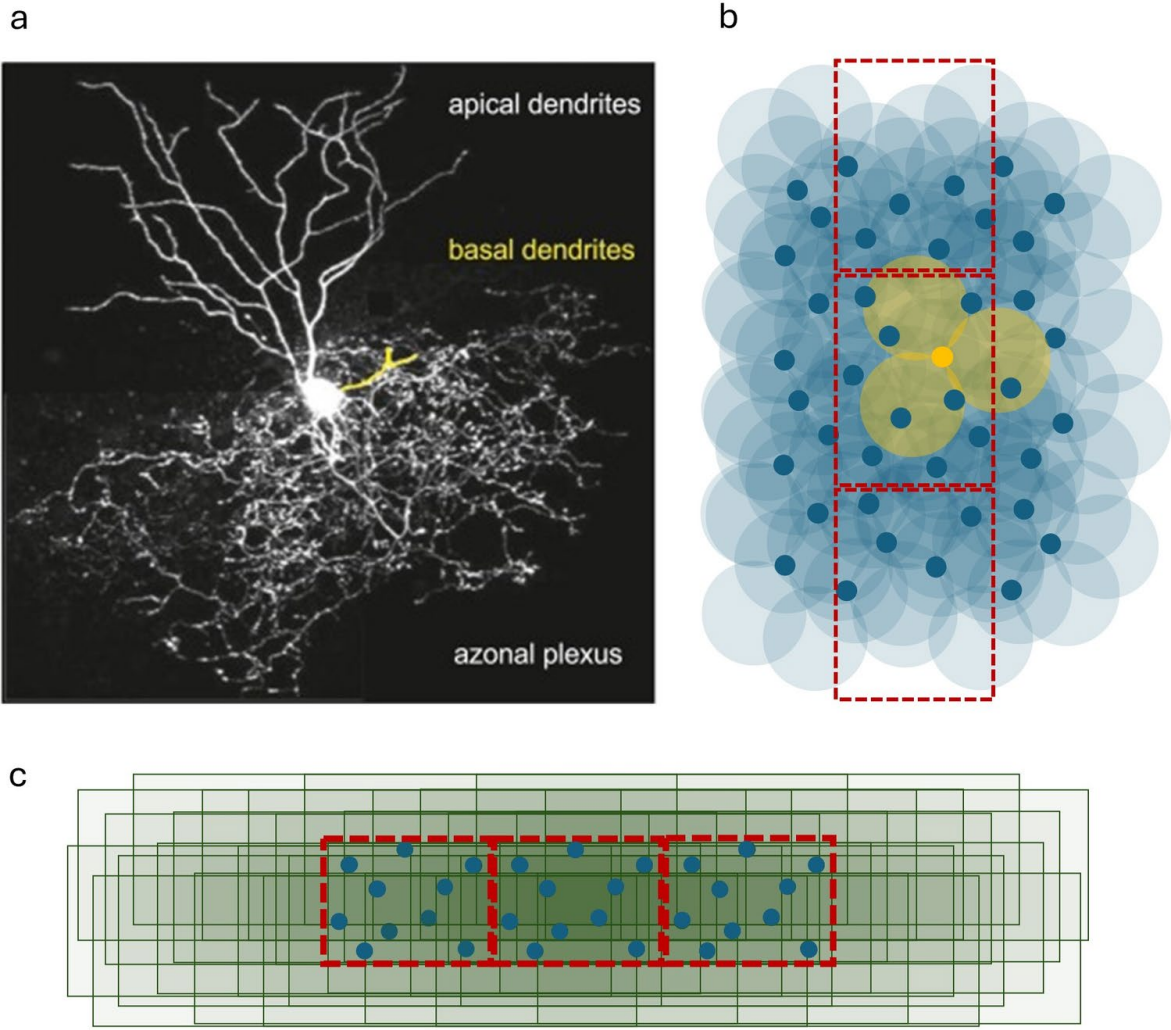
Schematic illustrating Golgi cell dendritic and axonal range. **a** Confocal microscopy reconstruction of a Golgi cell, reproduced with permission of Court Hull and Wage G. Regehr. **b** Schematic illustrating the range of Golgi cell apical dendrites, assuming 3 arise from the soma per cell and each dendrite is a columnar volume with a diameter of 100 μm, viewed from the cerebellar surface. Red rectangles are nominal field boundaries. Dark blue circles are Golgi cell somata, approximately to scale. The three pale yellow circles represent dendritic columns of a single cell. Any single circle intersects with more than 20 others. **c** Schematic illustrating convergence of axonal territories of the Golgi cell population of 3 fields onto the middle field. Each axonal territory is a transparent green rectangle with a blue circle in the middle representing the cell soma. The middle field is dark green: all territories either completely enclose the middle field or overlap with the majority of it

Gap junctions are formed of a plaque – a region where contact is made – uniformly peppered with ungated channels (typically some tens per plaque) [8]. Through these, charge seeks equilibrium, passing in the direction of the electrical and diffusion gradient. The number of gap junctions between two connected dendrites is in the range 1–9 [8, 10]. The range may partly reflect the fact that there is more overlap with nearer cells, because overlap of dendritic territories with nearer neighbours extends to a greater depth, so that contact can be along a greater length of connected dendrites. The strength of gap junction contact between two cells may therefore be a function of the distance between them [8]. Figure 1 includes an illustration of dendritic range.

The discharge of an action potential by a Golgi cell depolarises its electrically-coupled neighbours. Under anaesthesia, this entrains spike timing between coupled neighbours, causing oscillations. However, even sparse excitatory input to Golgi cell networks makes oscillations disappear [10], suggesting that, during behaviour, entrainment of spike timing is not the function of gap junctions.

We hypothesise that it is instead charge sharing in the locomoting animal, in motor circuits, so that charge becomes equalised between gap-junction-connected dendrites at a sustained but time-variable level. The level is accordingly proportional to the number of recently active inputs to a gap junction group. The duration of the integration window depends on how long perisynaptic ion channels are open after activation and the rate internal charge is cleared either passively or by leakage. Charge movement across small distances is extremely fast. Modest apical dendritic branching may aid the even spread of charge. Activation of a small number of synapses at any time means there is a lower probability of clustering of co-active synapses, so that – taken together – there is a relatively uniform electrochemical gradient across the dendritic membrane along the whole length of a dendrite.

### Golgi Cell Firing Rates Scale Proportionally with the Mean of Dendritic Charge

Golgi cells fire autonomously at a relatively modest rate [11] that is adjustable in both directions under input. This is seen, for example, both in the cat, in the paravermal cortex – 14.5 Hz tonically and 2-50 Hz on a treadmill [12] – and in the monkey vestibulo-ocular reflex, measured in the flocculus, 10-80 Hz [13].

We hypothesise that modulation of sustained dendritic charge has a likewise sustained effect at the Golgi cell soma. The effect is depolarisation and proportional to the mean of dendritic charge (passive dendritic charge transfer means it can also flow out of the soma, so there is a fast somatic response in both directions to fluctuation of dendritic charge). Somatic depolarisation is converted into a proportional firing rate – Golgi cell firing rates have a linear relationship with the amplitude of depolarising current [7].

### Inhibition of a Granule Cell is Proportional to the Mean of Golgi Cell Rates Afferent to a Glomerulus

As noted, inhibition of granule cells is almost exclusively via intraglomerular GABA spillover acting through non-synaptic GABA_A_ receptors [14] which are sensitive to the concentration of GABA [15]. It is unknown how many Golgi cells provide fine axon filaments that enter a single glomerulus – the simulation assumes 8–12, about 1/3 of the number in range. It has been proposed that, like Golgi cell dendritic signalling, there is a sustained build-up of glomerular GABA during behaviour [16] at an adjustable concentration controlled by Golgi cell firing rates [17]. Assuming spillover from a single afferent cell is proportional to its firing rate, spillover provides the physiological equivalent of averaging afferent rates.

In this way, we suggest, physiology makes inhibition a proportional function of the mean of Golgi cell firing rates that are received as convergent input to a glomerulus. Each of the (3–5, average 4) dendrites of a granule cell extends into a different glomerulus. A granule cell dendrite is about 15 μm long. Each glomerulus receives convergent innervation from a random sample of Golgi cells afferent to that location, so that each granule cell receives convergent inhibition of each of its dendrites from a different subset of Golgi cells. Unitary recordings confirm that a granule cell receives inhibition from multiple Golgi cells [18].

## The Golgi Cell Ensemble Computation

### The Distributed Probability of Active Inputs to a Golgi Cell Depends on the Fraction of Parallel Fibres that is Active (Fig 2 Column 1)

Granule cells receive input to the cerebellum from mossy fibres. The granule cell axon rises from the granular layer into the molecular layer where it divides in two. The two branches – parallel fibres – travel in opposite directions for about 3 mm [19, 20]. A Golgi cell accordingly receives contact from granule cells up to 3 mm away in both directions.

**Fig 2:**
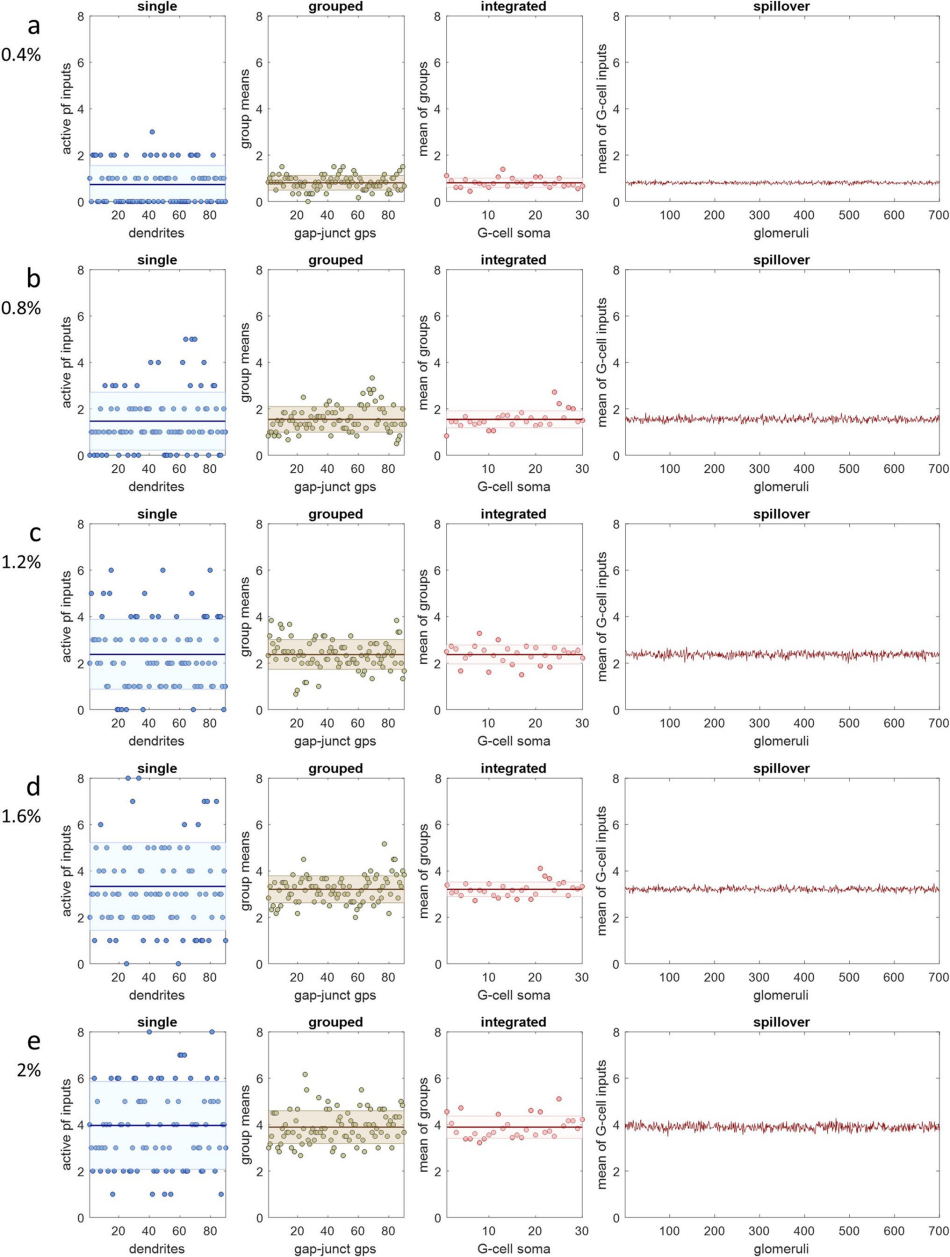
Simulation of the Golgi cell ensemble computation. The fraction of active parallel fibres in the general population is shown on the left of each row of panels. Column 1 Data represent the number of active inputs received by each of 90 Golgi cell apical dendrites (3 dendrites per cell x 10 cells per field x 3 fields). The number is randomly generated with a distributed probability derived from the percentage of parallel fibres that are active in the general population. Column 2 Each dendrite makes gap-junction contact on several others. The physiological number is unknown. We assume 6 (a single dendrite is in range of perhaps 4 times that number of its neighbours). In the behaving animal, the constant turnover of parallel fibre signals received by a group, combined with charge movement through gap junctions, causes dendrites to be in a state of sustained depolarisation. In the simulation, we represent dendrites as a matrix. The value at each index position is the average of a random sample (sample size 6) of surrounding index positions. Data are the sample means. Column 3 Column 2 data, representing dendritic states, were randomly divided into pre-assigned groups of 3, each representing a Golgi cell (with 3 apical dendrites where they emerge from the soma), and averaged, representing somatic integration (30 values, 10 per field). Column 4 The axonal territory of a single Golgi cell extends in both sagittal directions to fill 3 fields. Therefore, Golgi cells in a row of 3 fields (an ensemble) have convergent input to the middle field. Each field contains 700 glomeruli. A single glomerulus receives convergent input from a random sample of Golgi cells afferent to a field. We randomly sampled column 3 data 700 times, sample size 8–12 (the physiological convergence ratio is unknown, but all 30 Golgi cells are in range). Conclusion Ensemble physiology provides a plausible substrate to convert input into well-predicted output. Output is synchrony of inhibition of middle field granule cells which scales proportionally with the density of parallel fibre activity in the general population. Shaded areas in columns 1–3 show SD of the data. Precision of synchrony is inversely related to the density of active parallel fibres

Golgi cell apical dendrites radiate from the soma before turning towards the cerebellar surface. A single Golgi cell apical dendrite and branches may traverse a territory with a sagittal span of 100 μm, typically crossing the depth of the molecular layer to reach the pial surface, so that the territory of a single dendrite in the sagittal plane, the plane orthogonal to parallel fibres, is about 300 x 100 μm. An estimated 175,000 parallel fibres pass through a territory of that size, i.e., about half the estimated number that pass through a Purkinje cell territory [21]. If 0.4% are active, 700 are active; for 0.6% it’s 1,050, for 0.8% 1,400 and so on.

Active parallel fibres are pseudo-randomly dispersed among the general population – termed decorrelation – a consequence of the mechanism which converts mossy fibre signals into granule cell signals [22, 23]. As a result, each parallel fibre is at any time equally likely to be active with a probability that depends on the density of active cells in the general population. A Golgi cell therefore receives, at any moment, a randomly variable number of active inputs in a range given by a probability distribution.

An estimated 1,200 parallel fibres make contact on a single Golgi cell [24], or 1 in ~292. There is a probability therefore of 1/292 = 0.00342 that, of those parallel fibres which are active at any time, any particular one makes contact. Assuming three apical dendrites per cell (where they arise from the soma), and uniform density of active parallel fibres in the general population, the probability of contact on a single dendrite by *k* parallel fibres out of the subset that are active (a percentage, *j*, of the general population) is therefore given by

$$\frac{\left(\left(\frac{j}{100}\right)\ast 175000\right)!}{k!\left(\left(\left(\frac{j}{100}\right)\ast 175000\right)-k\right)!}\ast {\left(\frac{0.00342}{3}\right)}^k\ast {\left(1-\frac{0.00342}{3}\right)}^{\left(\left(\frac{j}{100}\right)\ast 175000\right)-k}$$

Table 1 shows the probabilities for the range *P*(*k*) > ~0.001 for *j* = 0.4–2 in steps of 0.2, where *k* is the number of active fibres that make contact. Rows are paired. The top row of each pair is the probability for active inputs to a cell, and the bottom row is the probability for active inputs to a single dendrite. Note: columns *k* = 11–21 only show row data – i.e., only the probability of *k* active inputs to a single cell, and not data a single dendrite – because the probabilities for a single dendrite are computationally (and therefore functionally) insignificant.

**Table 1:**
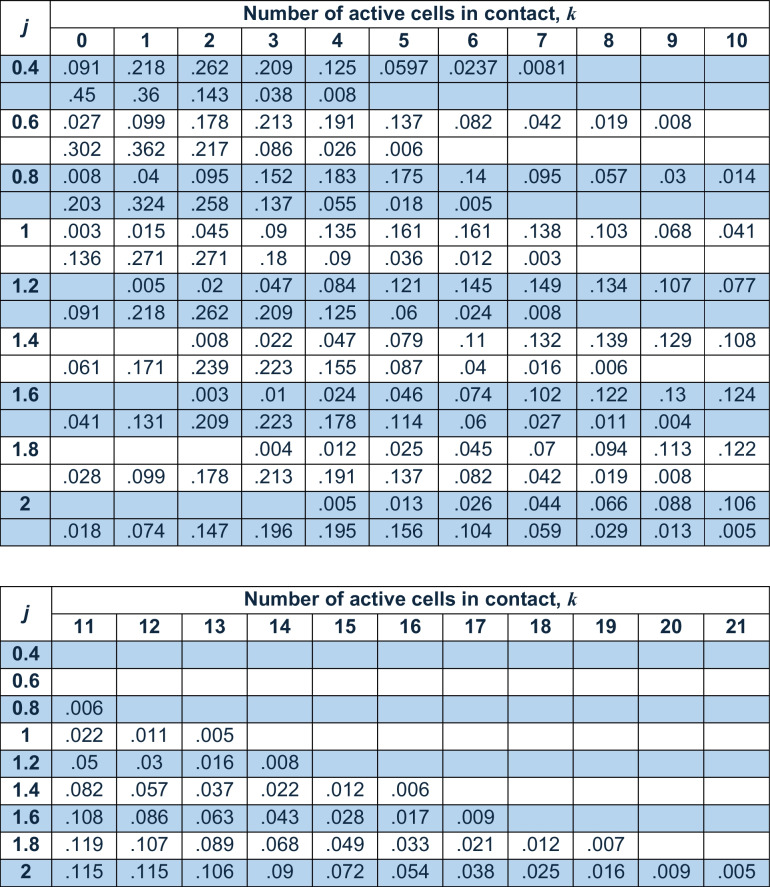
Distributed probability of active inputs to a Golgi cell and a single dendrite

Legend. Probability of apical dendritic contact by *k* active parallel fibres on a single Golgi cell and on a single Golgi cell apical dendrite, in the range *j *= 0.4–2% of parallel fibres active in the general population. Probabilities under 0.001 are not shown because they are remote and therefore lack a significant computational effect. In the range *k *= 0–10, there are two rows for each value of *j*. The probability of contact by *k* active parallel fibres on a single Golgi cell is shown in the top row of each pair. The probability of contact by *k* active parallel fibres on a single dendrite is shown in the bottom row. In the range *k *= 11–21, bottom rows are omitted because probabilities are all below 0.001

The distribution of probabilities is specific to the fraction of active parallel fibres in the general population. The simulation of the ensemble computation uses the distribution for each fraction to randomly generate the number of active inputs received by each of the apical dendrites of ensemble-grouped Golgi cells.

### The Computational Significance of Gap Junctions (Fig 2 Column 2)

#### Gap junction groups randomly sample a nominal distribution

The function of Golgi cell dendritic gap junctions is computational, we propose. Gap junction-connected dendrites share charge – in this section, ‘dendrite’ means a primary dendrite and any branches. A dendrite and the group of dendrites with which it is directly connected (which may not all be connected with each other) will be called a gap-junction group. The number of active inputs to a gap-junction group is in a larger (than a single dendrite) but still probability-restricted range. The average size of a gap-junction group is unreported but the number in any instance is not more than 15–20, the number of other dendrites in range.

Granule cells typically fire in short, high-frequency bursts of a few spikes. The length of bursts varies: 10–20 ms has been reported in adult cats [25] and 8–40 ms in rabbits [26]. As an example, a 15 ms burst at 300 Hz would mean a synapse that is activated by the signal receives 4–5 spikes at intervals of 3.33 ms. For parallel fibre signals density to be maintained at a stable state, therefore, mossy fibre signals must drive a steady turnover of granule cell signals.

Assuming the fraction of active parallel fibres is maintained at a stable level, there is a full turnover of signals (in the general population and therefore, on average, received by a group) in around the average duration of a granule cell burst. The mean rate of a full turnover of active inputs to a gap-junction group is independent of group size, but the rate that new signals are received is faster for a group than for a single cell. So grouped dendrites receive as a unit more smoothly sustained synaptically-mediated charge influx. Charge influx is sufficient for a build-up of dendritic charge during behaviour, so that a group is in a sustained state of depolarisation.

A group has unique membership and is defined by the group member which is directly connected with all the others by a chain of one link. Of course, two group members may also be connected indirectly, by a longer chain, but a group is defined by one link connections; note: a chain is a chain of dendrites, not cells, although they all belong to different cells. Each of the other group members defines, in turn, a (different) group with which it directly shares charge, and so on. As dendritic depolarisation is sustained, equalisation is ongoing.

The number of concurrent active inputs to each dendrite in a group is effectively a random selection from a pool of values given by a probability distribution which in turn reflects parallel fibre signals density and therefore the percentage of the general population of parallel fibres that are active. The pool itself is nominal – it has no tangible counterpart. The size of the range of values is the difference between the minimum and maximum number of active inputs with a more than negligible probability. Values occur in the pool – the sampled population – with a weighted distribution that depends on their probability.

So, the shifting number of active inputs to a gap-junction group provides, at any moment, the physiological equivalent of randomly sampling the nominal pool of values. Charge sharing by dendrites in a gap junction group provides the tangible counterpart of taking the sample mean (so: one mean per group). Anatomical overlap of groups means there can be more of them per Golgi cell and per unit volume. Sampling and taking the sample means generates a new population of values which are the input to the next step in the computation (following subsection).

Biology in this way simulates sampling with replacement. Replacement means, samples are independent – no sample has an effect or influence on the make-up of any other sample. If we were taking numbered balls out of a bag, they would be replaced between samplings. That has important consequences because it causes the frequency distribution of the sample means to contract around its mean, which is the same as the mean of the sampled distribution, and also causes it to be normally distributed, or reliably attracted to a normal distribution. The strength of the attraction – that is, the closeness of the new distribution to normal – depends on the number of samples (the number of gap junction groups) and sample size (the number of dendrites in a group). It also depends on the size of the range of the sampled distribution, the nominal pool of values.

The results are shown in Fig. 2 column 2. Each row shows the results, from top to bottom, for an increasing fraction of active parallel fibres.

#### Computationally-Relevant Physiological Parameters

To quantify these ideas, we work with ‘fields’. A field is a 200 μm x 150 μm (sagittal by mediolateral) area of the granular layer, the average size of mossy fibre branch endings, which form a cluster of terminals [27]. Fields are not anatomically defined but are nominal divisions of the granular layer, which is an anatomically unbroken carpet of cells.

The Golgi cell axonal tree branches repeatedly at right angles to form a dense plexus which fills the granular layer vertically and extends sagittally in both directions (mean range 650 +/- 179 μm by 180 +/- 40 μm [in mice: [28]). The plexus of a single cell fills a sagittal row of about three fields, and Golgi cells in a three-field row all have convergent input to the middle field: an ‘ensemble’. All Golgi cells inhibit substantially the whole of the middle field so that each glomerulus in the middle field is in range of all of them.

An ensemble is defined as the group of Golgi cells afferent to a field, which is the Golgi cell population of 3 fields. The number of gap junction groups in an ensemble is equal to the total number of apical dendrites of that population. As noted, there are 2–4 apical dendrites where they emerge from the Golgi cell soma [1]. We assume 3. The population of apical dendrites in 3 fields is therefore 90, assuming a ratio of Golgi cells to Purkinje cells of 2:1 (an estimate for monkeys and cats; in man it is lower and in rats higher [29]), and 5 Purkinje cells per field.

We take it that the horizontal dendritic range of a Golgi cell has a radius (centred on the soma, viewed top down from the cerebellar surface) of around 100 μm, so by extending their dendrites towards each other, cells whose cell bodies are 200 μm apart can make dendritic contact.

The range of a single dendrite is smaller. If a dendrite is viewed as a columnar volume with a diameter of 100 μm, it is in range of over 20 neighbours (assuming a 2:1 ratio of Golgi cells with Purkinje cells [29]), although overlap with some is modest (Fig 1). It is unknown what fraction of those make contact.

At any moment, an ensemble takes simultaneous samples of a nominal population of values. The number of samples is equal to the number of gap junction groups. Sample size is equal to the unknown and doubtless variable number of dendrites per group. We assume 6 (a single dendrite is in range of perhaps 4 times that number of its neighbours), intended to be hypothesis neutral. Group means are taken ‘simultaneously’ (so that the calculations do not receive an arbitrary effect of the order in which they are performed).

Taking the mean of single-link groups is a simplification. Physiological charge sharing is by chains of more than one link, and by more than one chain between any two points. Links are not a chain but a web; each dendrite is ultimately connected to all its neighbours by multiple pathways. We make two observations. First, a longer chain is a weaker influence. The individual influence of a single dendrite is dramatically weakened even by adding a single link. Second, web-sharing would increase the attraction to ensemble-wide equalisation. As we claim this is a function of charge sharing, it would be hypothesis favourable.

### Averaging of Dendritic Charge by Somatic Integration (Fig 2 Column 3)

We allocated the group sample means to pre-assigned groups of 3 and again took the means, representing somatic integration, generating 30 data points (30 somata, 10 per field). Averaging causes somatic depolarisation of ensemble-grouped Golgi cells to converge towards alignment.

This step incorporates conversion of somatic depolarisation into a proportional firing rate. Firing rates have a linear relationship with the amplitude of depolarising current [7]. As noted, information transitions through several biophysical forms in its passage through an ensemble. We do not change the scale of measurement; we use a normalised scale to quantify data at all steps. Accordingly, at this step, Golgi cell somatic charge and firing rates are represented by the same data set.

### Middle Field Glomeruli Randomly Sample Golgi Cell Rates (Fig 2 Column 4)

Glomeruli in the middle field each receive innervation from a random sample of Golgi cells in a 3-field row, and therefore randomly sample firing rates of Golgi cells afferent to the field. There are an estimated 700 glomeruli per field, derived either from an estimate of the number of granule cells per field (8,750) and a mossy fibre terminal to granule cell divergence ratio of 1:50 [30, 31] (estimates vary [32]), or from estimates that 100 mossy fibres innervate a field (as a proportional fraction of the number that innervate a larger area) and mossy fibres terminate in an average of 7 terminals [27].

This physiological architecture is designed for a computational effect. We took 700 random samples of the column 3 somatic data and plotted the sample means, sample size 8–12, individually generated for each sample, around a third of Golgi cells that innervate the middle field. Biology again simulates random sampling with replacement – that is, each glomerulus can receive innervation from any subset of Golgi cells afferent to the field, regardless of the subset that innervate any other glomerulus – and the significance is again that this has statistically reliable consequences for the frequency distribution of the sample means. The consequences are that the distribution contracts again around the mean, on which it is centred, and which is the same as the mean of the sampled distribution. Physiologically, that means spilled-over GABA concentration is tightly aligned between glomeruli and scales proportionally with the mean of Golgi cell rates.

So: we find that input to an ensemble (in the simulation, a randomly generated number of active inputs to apical dendrites generated with the Table 1 probability distribution) is converted to well-predicted output (proportional inhibition of granule cells that is predicted by input values, and which is in a narrow range). We conclude that ensemble physiology is a plausible substrate to implement a computational conversion.

## Accuracy and Precision of the Ensemble Conversion are Inversely Related to the Percentage of Active Parallel Fibres (Fig 3)

We next investigated the effect of varying the percentage of active parallel fibres on the accuracy and precision of the ensemble conversion. Precision refers to the spread of 700 values returned as output of the conversion which each represent the mean of Golgi cell rates received by a glomerulus. This is the output of a single ensemble to a single field. Accuracy refers to reproducibility – if we take the mean of those values, is it always the same if we run the test many times, and if not, how tightly grouped (or not) are the results?

**Fig 3:**
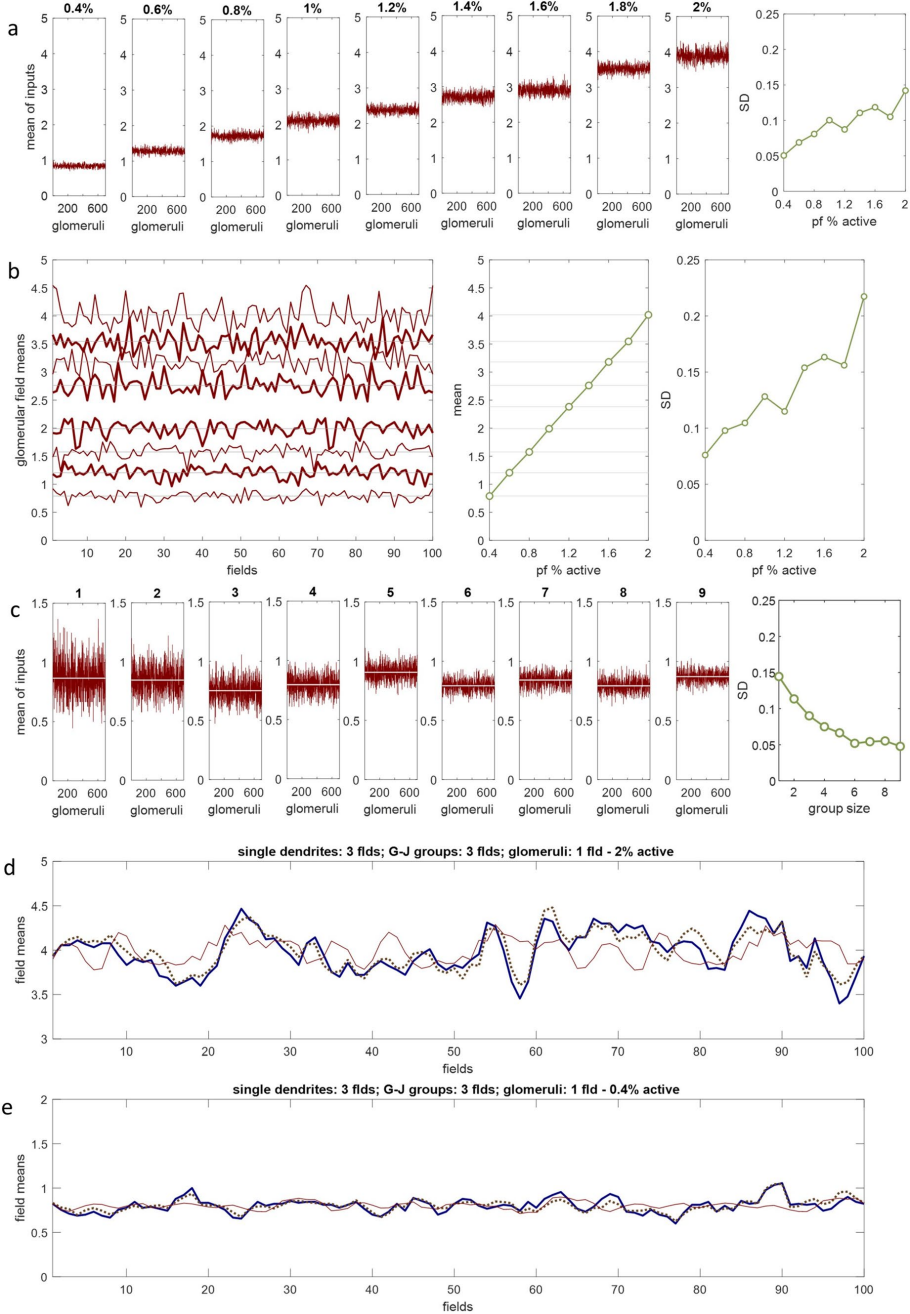
Golgi cell ensemble conversion: precision and accuracy. **a** and **c** show output of an ensemble to a single field; **b, d** and **e** show results for 100 fields. All red data are derived in the same way as column 4 in Fig 2. The percentage of active parallel fibres impacts precision and accuracy of output. Row a shows the impact on precision. Data are tightly grouped with 0.4% active (high precision; narrow red band). Increasing the percentage of parallel fibres that are active in the general population causes a progressive loss of precision from left to right. SD of the data in each panel is shown in the rightmost panel. Row b Left panel: We ran the simulation 100 times, first with 0.4% of parallel fibres active, then 0.6%, then 0.8%, 1%, 1.4% 1.6%, 1.8% and 2% (bottom to top). Each data point is the mean of 700 values generated for a field, so each data set contains 100 values, 1 mean per field. Centre panel: We took the mean of each data set. There is a linear relationship of with the percentage of active parallel fibres. Right panel: SD of each data set. Higher SD equates to lower accuracy (reduction of reliability). Higher levels of parallel fibre activity make ensemble output less reproducible (also seen as an increase in the vertical range of the data in the left panel from bottom to top). Row c Precision is also affected by gap-junction group size (higher precision with larger groups) and the convergence ratio of Golgi cells on a glomerulus (higher precision with a higher ratio, not shown). Group size is shown above each panel. Apart from varying group size, data are obtained in the same way as the left panel in row (a) but y axes have a shorter range. SD of each data set is shown on the right. There is a rapidly diminishing return of increasing group size, and almost no return of increasing it above 6. Rows d and e We ran the ensemble conversion 100 times sampling the same mossy fibre distribution. Blue data: the mean number of active inputs to a single Golgi cell dendrite (ensemble); dotted line: the mean number of active inputs to a gap-junction group (ensemble); thin red line: the mean of output data (received by the middle field). Data are accordingly derived the same way as columns 1, 2 and 4 of Fig 2 respectively, except that values for each field are averaged (so red data are derived the same way as row b). Data representing somatic integration are omitted as they exactly follow the dotted line. The dotted line closely tracks the blue line but red data vary independently, except in 2 parameters, mean and SD. Control by input of output is only of these parameters. However, the percentage of active parallel fibres very powerfully affects SD (**d**: 2% active; **e**: 0.4%). Low SD here represents high accuracy/reproducibility of the conversion of the active fraction into proportional inhibition of granule cells (in addition to high precision at low density, seen in (**a**) and (**b**))

We found that increasing the fraction of active parallel fibres reduces precision (Fig 3a). The reason is that it increases the size of the range of the number of co-active inputs received by a dendrite with a more than negligible probability, and to accommodate that, probabilities are smaller and so become nearer equal, so that there is a wider spread of nearer-to-equally likely outcomes. Less focused input drives less focused output.

We also tested the effect of gap junction group size on precision (Fig 3c). Large group size increased precision but there was an unexpectedly strongly diminishing return and almost no extra precision over a group size of 6. This would suggest there is little advantage in having apical dendrites which radiate further from the cell body, or which branch more, to allow them to contact more neighbours.

We next tested accuracy by simulating 100 fields. The results are shown in Fig 3b. Increasing the percentage of active parallel fibres causes a progressive loss of accuracy. The reason is the same as the reason there is loss of precision: it increases uncertainty, so there is a larger range of outcomes with a significant probability. No other tested parameters affected accuracy.

This result predicts sparse code because density should be attracted to a stable state if it self-regulates. The self-selecting state of a closed system that self-regulates is stability.

## Discussion

This paper describes a mechanism which feasibly converts parallel fibre signals density – the percentage of the general population that are active at any time – into a proportional adjustment of inhibition of granule cells. The aim of the paper is to explain neurophysiology as the implementation of a computational conversion which can account for the evidence.

The matrix-like anatomy of the cerebellar cortex has long looked likely to be related to its function. Golgi cells send a small number of modestly branching axons into the molecular layer which radiate in all directions from the soma. Their morphology is strikingly different from other cell types in the molecular layer, which are very severely flattened in the plane orthogonal to parallel fibres. Some hundreds of thousands of parallel fibres pass through the Golgi cell dendritic territory, of which around 1,200 make contact. If, say, 1% of parallel fibres are active at any time, active inputs to a Golgi cell represent 0.00343% of the general population, from which they are drawn at random. Granule cells fire in short bursts. A Golgi cell receives, at any moment, a randomly variable number of active inputs in a range given by a probability distribution. The set that are active, and the number, is in a state of flux. The rate of change depends on the rate of turnover of parallel fibre signals in the general population.

Our aim is to explain how parallel fibre input to Golgi cells codes anything. We make a case that physiology provides the automated equivalent of a count of active inputs to a functional group of Golgi cells. We describe the form and transmission of information as a chain of biophysical events and propose that cell morphology and ensemble architecture have a computational effect, which we computer simulate.

This has the features of being a mechanism of neuronal ‘computation’ that is not synaptic plasticity, explaining the functional design of a Golgi cell network, and proposing a form of group code. An important difference with other network models (that we know about) is that information is *conserved* in transit through the network – the function is to change the form of the code and not to alter the information.

A Golgi cell ensemble is the population of a row of three fields at right angles to parallel fibres. Ensembles are not anatomically bounded but a functional division of an unbroken carpet of Golgi cells. Unlike microzonal organisation – functional organisation of the cerebellar cortex into long thin strips – ensembles overlap anatomically. An ensemble is simply a minimum functional size. That is, they represent the minimum resolution of parallel fibre information (coded in the form they are equipped to read). Firing of ensemble-grouped cells is accordingly a collective code notwithstanding that functional ensembles are not even functionally segregated.

In the traditional cerebellar learning model – the supervised learning model [33] – the functional rationale for sparse code is that it increases the number of patterns that can be stored as synaptic changes. There are other reasons for a low and stable level of parallel fibre activity.

The reason for a low level is the energy cost. This is an especially acute concern for granule cells. There is a high energy cost of neural signalling, much of it on pumping Na^2+^ ions back out of the cell after an action potential (even after revising the cost downwards [34]). Granule cells account for over half the cells in the brain. They are unmyelinated, so the cost of a spike is high (because it is not reduced by cable transmission between unsheathed nodes). If granule cells were continuously active, or a large fraction was typically active, the cost would be very high. Firing in short bursts by a low fraction makes a large saving.

The argument for a stable level is the general requirement to eliminate non-functional parameters. Non-functional parameters are variables contained in collective activity that code nothing. Noise competes for influence with data-bearing parameters. The problem is compounded if there is a series of noisy relays. As far as is known, apart from a role in self-regulation, the proportion of active parallel fibres is not code, so that a fluctuating proportion would be noise.

Do Golgi cells receive other inputs that would interfere with the computational conversion? Contact has been reported to be made on Golgi cells by a number of cell types. However, contrary to early reports, neither Purkinje cells nor climbing fibres contact Golgi cells [35], who give references], and (if they exist) only a modest minority of inhibitory inputs to Golgi cells are from molecular layer interneurons [36], which generate weak synaptic currents [37], consistent with either extremely weak or lack of innervation [38]. There is conflicting evidence whether Golgi cells make inhibitory synaptic contact on each other [10, 38].

There is a strong effect on Golgi cell firing from mossy fibres which make direct contact on Golgi cell basal dendrites [18]. It has been argued that this may in fact be the dominant controller of Golgi cells, and also the primary regulator of the fraction of active parallel fibres [23]. In this view, homeostatic regulation by feedback via parallel fibres has a relatively minor modulatory effect. The light touch is functionally ‘intentional’ so that it does not interfere with local control.

Golgi cells also receive significant synaptic contact from Lugaro cells [37], inhibitory interneurons. The function of Lugaro cell contact on Golgi cells is unknown. However, inhibition is driven by serotonergic innervation of Lugaro cells from the dorsal raphe [39], and not narrowly targeted, suggesting it is involved in external regulation of general system activity. It may regulate circadian up and down states.

There are neurochemically-defined subpopulations of Golgi cells [40] but data are very limited and any functional significance is unknown. An autonomously silent inhibitory projection from the deep cerebellar nuclei to the cerebellar cortex targets a subgroup of Golgi cells that are spontaneously active and express neurogranin, but not GlyT2 [40]. It is not clear what activates this pathway although there is indirect evidence for climbing fibre collaterals [41, 42].

So, some inputs to Golgi cells that were thought to exist probably don’t, and some others may, but with an unknown function or effect. It is also unknown whether and to what extent Golgi cell apical dendritic signals reflect granule cell firing rates. It is possible that parallel fibre synapses are in some way adapted to mitigate an effect. If not, we cannot rule out an effect on Golgi cell firing of parallel fibre rates in tandem with the proposed rolling count of active inputs.

## Data Availability

No datasets were generated or analysed during the current study.

## Footnotes

1 Either spontaneous or chemically induced in young rat slices.
